# Wang procedure for secondary thoracic deformity after simultaneous operation of sternal cleft with congenital heart disease

**DOI:** 10.1093/jscr/rjac574

**Published:** 2022-12-09

**Authors:** Wenlin Wang, Weiguang Long, Yang Liu, Bin Cai, Juan Luo

**Affiliations:** Department of Chest Wall Surgery, Guangdong Second Provincial General Hospital, Guangzhou, China; Department of Chest Wall Surgery, Guangdong Second Provincial General Hospital, Guangzhou, China; Department of Chest Wall Surgery, Guangdong Second Provincial General Hospital, Guangzhou, China; Department of Chest Wall Surgery, Guangdong Second Provincial General Hospital, Guangzhou, China; Department of Chest Wall Surgery, Guangdong Second Provincial General Hospital, Guangzhou, China

## Abstract

Sternal cleft is a rare thoracic deformity, often associated with congenital heart disease, which requires simultaneous treatment. There are many methods for treatment of sternal cleft, the early effect may be satisfactory, but the late effect is rarely reported. We performed a simultaneous operation on a patient with sternal cleft and ventricular septal defect, but the patient developed secondary depression deformity after the operation. We used Wang procedure to correct it and got satisfactory results.

## INTRODUCTION

Sternal cleft is a rare chest wall deformity, often associated with congenital heart disease [1–4]. Both lesions require simultaneous surgery [4]. There are many surgical methods for sternal cleft with an early postoperative effect, but secondary chest wall deformity may occur in the late stage and needs to be corrected. Recently, we performed surgical treatment on a patient with secondary deformity after simultaneous operation of sternal cleft and congenital heart disease, and achieved satisfactory results.

## CASE REPORT

**Figure 1:**
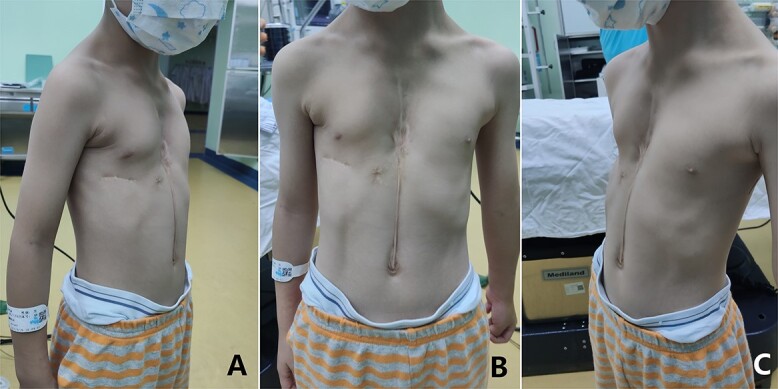
Appearance of chest wall before operation.

**Figure 2:**
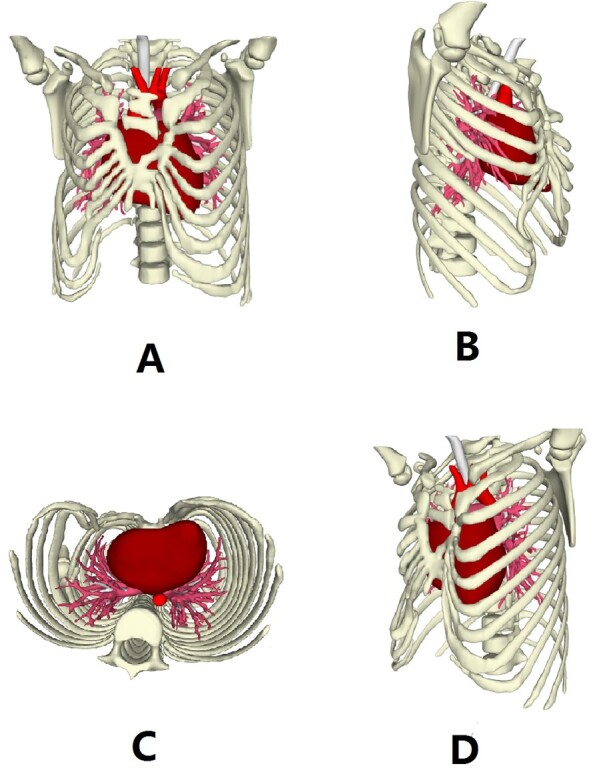
Three dimensional reconstruction images of the chest wall.

**Figure 3:**
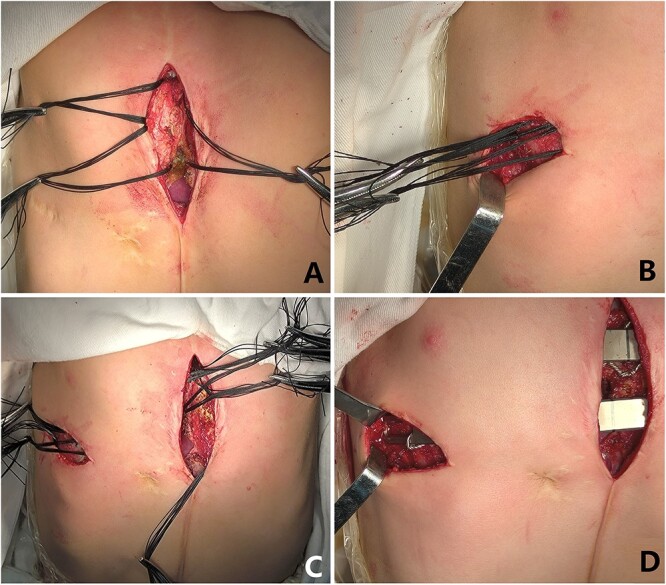
(**A**) Median operative field; (**B**) Right chest wall operative field; (**C**) The steel wire guiding lines were placed completely; (**D**) The depressions were completely eliminated with two steel bars.

**Figure 4:**
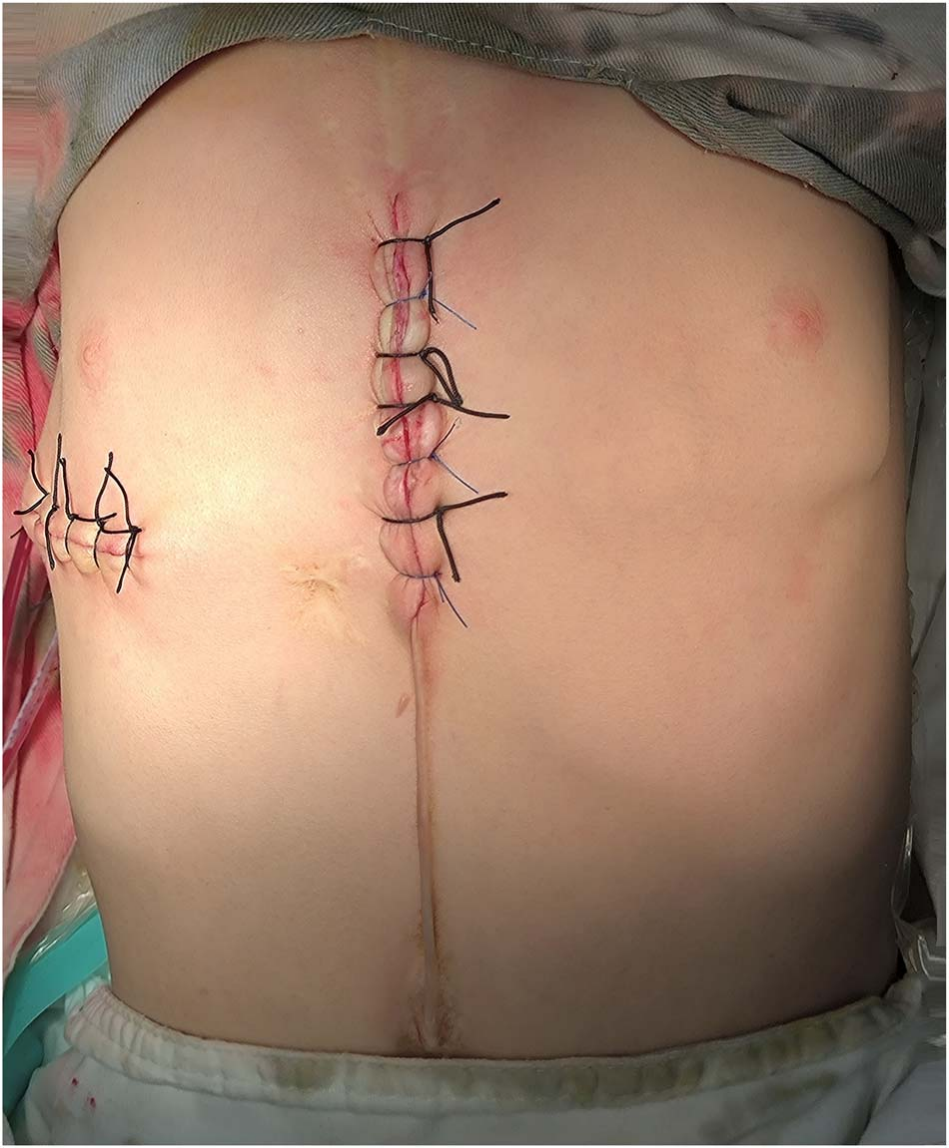
Appearance of chest wall after operation.

The patient, a 5-year-old boy, was admitted to our hospital for surgery due to thoracic deformity. Shortly after his birth, he was diagnosed as sternal cleft with ventricular septal defect. We performed surgical treatment for him in 1 month after birth. We first implemented correction of ventricular septal defect through the median sternotmy under cardiopulmonary bypass, and then repair the sternal cleft using autogenous rib grafts, which were cut from the fifth and sixth ribs on the right chest wall. The rib grafts were placed horizontally in the middle of the two sternal flaps and fixed to them. After operation, the patient recovered smoothly, no residual defect was found in the heart, but the anterior chest wall gradually became deformed. With the increase of age, the deformity gradually worsened. In order to correct the deformity, the child was recently admitted to our hospital for surgery. Physical examination before operation showed that there were scars on the front and right side of the chest wall, and there were depressions in the middle of the front and the right chest wall (Fig. 1). The preoperative imaging examination showed that the chest wall was sunken in the middle, both sides were protrusive, the right rib arch was sunken in the middle, and the right fifth and sixth ribs were partially defective (Fig. 2). No abnormality was found in the heart. The operation was performed under general anesthesia. The method we used was Wang procedure ([5]; Fig. 3). Two incisions were performed at the scar of the median and side chest wall respectively to expose the bone structures at the bottom of the depressions. Several steel wire guiding lines were placed across the bone structures at the bottom. Two tunnels were made on both sides of the chest wall from the two incisions, which were located between the bone structures and the muscles. After two steel bars were inserted into the tunnels successively, the steel wires were placed with the guiding lines. The bone structures at the bottom of the depression were lifted with steel wires and fixed on the steel bars, and the depressions were completely eliminated. After the drainage tubes were placed in the operation field, the incisions were closed, and the operation was completed (Fig. 4). The operation was smooth without complications. After operation, the thoracic deformity disappeared and the appearance returned to normal. The patient was discharged ten days after operation.

## DISCUSSION

Sternal cleft is a rare chest wall deformity, with very few case reported worldwide [1–3]. Since the integrity of the thorax is damaged, which may affect the function of chest wall and lung, surgery is required. This deformity often associated with congenital heart disease, once confirmed, surgery should be completed at the same time [4]. Cardiac surgery can be performed routinely through the median sternotomy, but there are two surgical methods for sternal cleft, namely, primary closure [1, 2] and reconstruction [3].

Primary closure is relatively simple, whereas reconstruction requires the use of special materials, which include autologous and synthetic materials [3]. In this patient’s surgery, we used autologous ribs. Although the early effect was satisfactory, but secondary deformity appeared in the late stage. The deformity not only came from the vicinity of the sternal cleft, but also from the chest wall where the ribs were cut, forming a complex thoracic deformity. This kind of deformity has not been reported in the previous literature.

The patient’s deformity is mainly depression, which mainly includes two parts: one part is located in the middle of the anterior chest wall, and the other part is located in the lateral chest wall. We used Wang procedure to perform the correction. The procedure is a minimally invasive operation for pectus excavatum [5]. However, recent studies have found that this kind of procedure can be used in the surgery of all depression deformities [6–8]. Since the steel bar is placed on the top of the depression, the depression can be eliminated when the bottom structures of the depression are lifted and fixed on the steel bar. The greatest advantage of this procedure is safety and simplicity. Because Wang procedure has the principle of template plastic surgery [9], the effect is also satisfactory.

The deformity of this patient is secondary depression. Because of serious adhesion between the chest wall and the heart, if Nuss procedure was used, there would be a greater risk [8]. In contrast, since Wang procedure is completed on the outer surface of the bone structure, it is safer and an ideal choice [8].

## CONFLICT OF INTEREST STATEMENT

None declared.

## FUNDING

None.
